# Artificially Sweetened Beverages Beyond the Metabolic Risks: A Systematic Review of the Literature

**DOI:** 10.7759/cureus.33231

**Published:** 2023-01-01

**Authors:** Tomas Escobar Gil, Juanita Laverde Gil

**Affiliations:** 1 Medicine, Universidad CES, Medellín, COL; 2 Medicine, Universidad CES, Medellin, COL

**Keywords:** artificial sweetener, diet soda, endocrinology, nutrition, psychiatry

## Abstract

We carried out a review of the available literature on the effects that artificially sweetened beverages (ASBs) such as diet soda (DS) have on health, particularly those not related to incident diabetes mellitus, obesity, and metabolic syndrome.

A search of scientific articles was carried out using 11 different databases: PubMed, Cochrane, LILACS, MEDLINE Ovid, JAMA Network, IBECS, Cumed, Scopus, SciELO, MEDLINE-EBSCO, and Taylor & Francis Online. Articles published in the last 10 years were considered, considering cross-sectional studies, retrospective or prospective cohort studies, case-control studies, and randomized controlled clinical trials. Only articles in Spanish or English were considered using the MeSH (Medical Subject Heading) and DeCS (Descriptores en Ciencias de la Salud) terms, including “Diet soda,” “Health,” “Artificial sweetener,” “Gaseosa sin azúcar,” “Refresco sin azúcar,” and “Salud.” Additionally, Boolean operators “AND” and “Y” were used.

A total of 1,323 articles were obtained in the initial search, of which 21 main ones were selected for review, which included the topic of DS consumption and explored the health consequences that it poses on different organs.

The question of whether ASBs such as DS are a preferred substitute is becoming more and more important in terms of public policy due to mounting evidence of the potential negative health effects of their excessive consumption. This systematic review, the first of its kind to our knowledge, sheds light on how excessive DS consumption can affect multiple organ systems, and associations have been made to mental health burden, delays in child neurodevelopment, cardiac remodeling, worsening retinopathy in diabetics, incidental end-stage renal disease, non-Hodgkin's lymphoma and multiple myeloma in men, rheumatoid arthritis in women, hip fractures, dental erosion, increases in breath alcohol concentration when used in alcoholic beverages, and accelerated cell aging. Further studies should delve further to understand the pathophysiologic mechanisms of these associations.

## Introduction and background

Increased advertising and usage of artificial sweeteners are a consequence of rising concerns over the harmful health effects of sugar consumption during the past three decades [1]. In fact, the global diet soda (DS) market is expected to grow at a compound annual growth rate of 3.2% from 2019 to 2025 to reach US$ 5.17 billion by 2025 [2]. Although the need to reduce the consumption of sugar-sweetened beverages (SSBs) is widely acknowledged due to known and well-studied metabolic risks, the question of whether artificially sweetened beverages (ASBs) such as DS are a preferred substitute is becoming more and more important in terms of public policy due to mounting evidence of the potential negative health effects of their excessive consumption [3].

The first artificial sweetener, saccharin, was originally synthesized in 1879 by Remsen and Fahlberg [4]. Aspartame was then discovered in 1965 [4]. Other artificial sweeteners like sucralose, acesulfame K, neotame, Stevia (rebaudioside A), and tagatose are newer [4]. These substances are all sweeter than table sugar and have no nutritious properties; this implies they provide no calories.

Recent studies have uncovered that ASBs - much like SSBs - are linked to increased abdominal circumference, incident diabetes, and cardiovascular events [1,5-12]. These findings have been controversial nevertheless, as other authors have not found the same metabolic associations [13-15]. It has been determined by consensus that excess consumption of these drinks should be avoided because their impact on health beyond the metabolic aspects is unknown and is yet to be studied in depth [3], which brings us to the question posed in the present study. What lies beyond the comparisons of ASB and SSB in terms of hormonal and metabolic consequences? What other organs and systems could be affected by artificial sweeteners? Have other risks been assessed? These are the inquiries that prompted the authors to delve further and examine the literature in pursuit of preliminary answers.

## Review

Materials and methods

**Figure 1:**
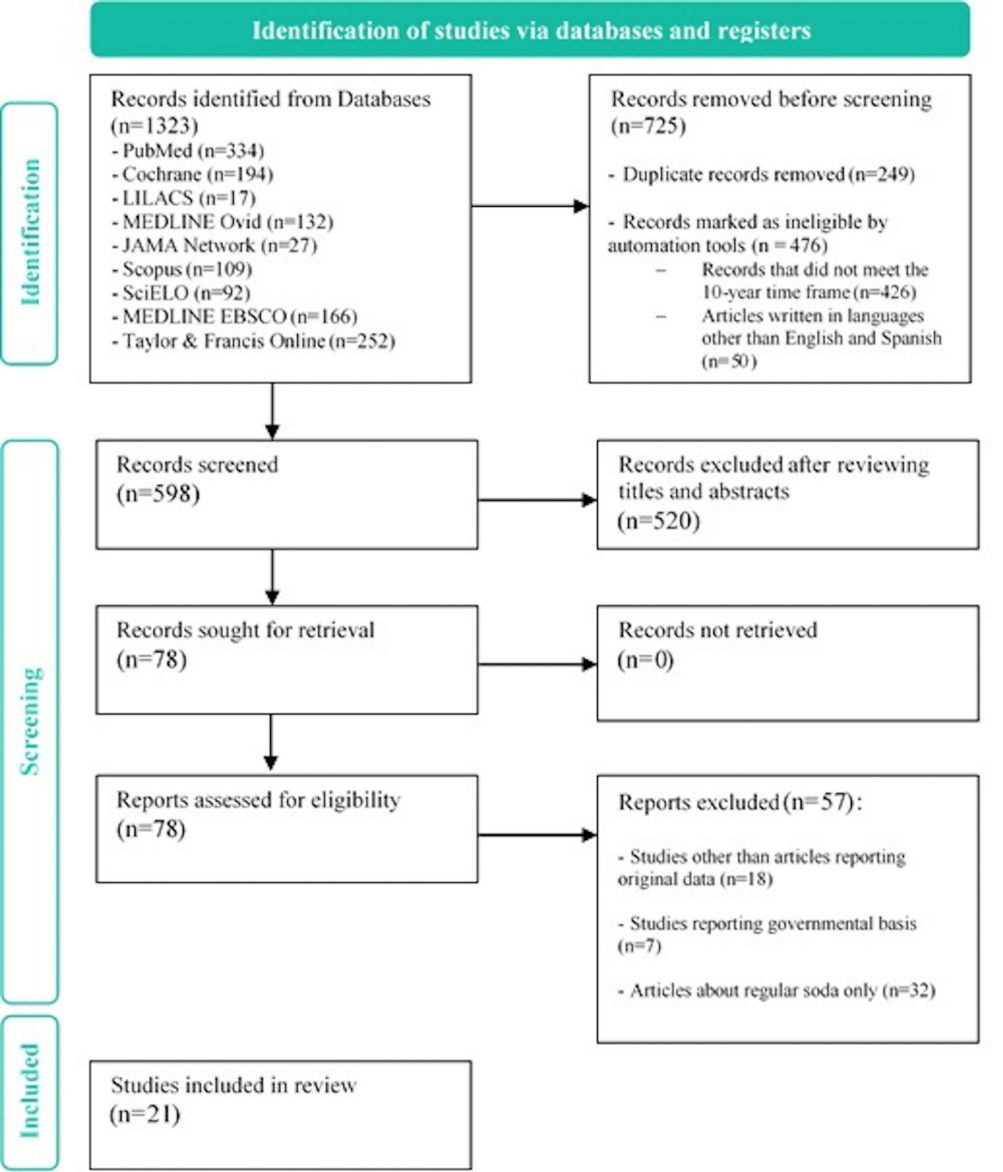
Flowchart for article selection.

A detailed literature search was conducted using the Critical Appraisal Skills Programme (CASP)/CASPe guidelines as a model for the design of this review [16,17], and the Preferred Reporting Items for Systematic Reviews and Meta-Analyses (PRISMA) statement for reporting systematic reviews was used for the design of the flowchart (Figure 1) [18]. Information was gathered from 11 databases: PubMed, Cochrane, LILACS, MEDLINE Ovid, JAMA Network, IBECS, Cumed, Scopus, SciELO, MEDLINE-EBSCO, and Taylor & Francis Online. The following MeSH (Medical Subject Heading) and DeCS (Descriptores en Ciencias de la Salud) terms were used: “Diet soda,” “Health,” “Artificial sweetener,” “Gaseosa sin azúcar,” “Refresco sin azúcar,” and “Salud.” Additionally, Boolean operators “AND” and “Y” were used. Only articles published between 2012 and 2022 were considered for revision, and filters were applied to obtain results in English or Spanish only and were then filtered by title and abstract.

Regarding the chosen study types and the applied inclusion criteria, only those with original data were included, selecting those that evaluated the relationship between ASBs and their impact on health variables. Only studies on humans were considered for analysis. Cross-sectional studies, retrospective or prospective cohort studies, case-control studies, and randomized clinical trials were included. Systematic review articles, mini-review articles, meta-analysis articles, opinion articles, letters to the editor, and complete books were excluded.

Among other exclusion criteria were studies about sweetened beverages only (i.e., regular soda), governmental or institutional efforts to reduce soda consumption, taxes on ASBs, economic studies regarding soda consumption, or specific approaches that did not include direct health impacts such as artificial sweeteners in breast milk or assessment of fertility. We decided to limit the amount and ultimately exclude most articles about incident diabetes, obesity, and hormonal changes, as these subjects have already been studied in depth and findings remain controversial, as described in the introduction. The main goal of our review was to explore beyond these known risks. The full text was only evaluated for articles that met all inclusion criteria. This process can be found in Table 1 and Figure 1.

**Table 1:** Search results in databases. MeSH: Medical Subject Heading; DeCS: Descriptores en Ciencias de la Salud.

Database	MeSH and DeCS terms used	Number of articles
PubMed	“Diet soda” AND “Health” “Artificial sweetener” AND “Health”	334
Cochrane	“Diet soda” AND “Health” “Artificial sweetener” AND “Health”	194
LILACS	“Gaseosa sin azúcar” Y “Salud” “Refresco sin azúcar” Y “Salud”	17
IBECS	“Gaseosa sin azúcar” Y “Salud” “Refresco sin azúcar” Y “Salud”	0
MEDLINE Ovid	“Diet soda” AND “Health” “Artificial sweetener” AND “Health”	132
Cumed	“Gaseosa sin azúcar” Y “Salud” “Refresco sin azúcar” Y “Salud”	0
JAMA Network	“Diet soda” AND “Health” “Artificial sweetener” AND “Health”	27
Scopus	“Diet soda” AND “Health” “Artificial sweetener” AND “Health”	109
SciELO	“Gaseosa sin azúcar” Y “Salud” “Refresco sin azúcar” Y “Salud”	92
MEDLINE EBSCO	“Diet soda” AND “Health” “Artificial sweetener” AND “Health”	166
Taylor & Francis Online	“Diet soda” AND “Health” “Artificial sweetener” AND “Health”	252

Results

A total of 1,323 records were identified through database searching. After excluding duplicates, records that did not meet the 10-year time frame and articles written in languages other than English and Spanish, and reviewing titles and abstracts, 78 were selected for full-text review. Of the latter, 57 were removed by exclusion criteria. Therefore, 21 papers were included in the systematic review; these comprised 14 cohort studies [19-31], five cross-sectional studies [32-36], a crossover study [37], a case-control study [38], and a randomized controlled trial [39].

Two researchers individually assessed each publication. Most of the participants in the papers under consideration were female [19,22,24-33,35,36]. Regarding the health consequences of DS consumers, the following were studied: mental health [20,32,36,38], child neurodevelopment [21], cardiometabolic impact [1,19,29,31], diabetic retinopathy [34], urogenital implications [26,28,37], carcinogenesis [30], fatty liver disease [25,27], autoimmune disease [24], hip fractures [22], and other health impacts [23,33,35,39]. After evaluating each article, 11 records, out of the total analyzed, demonstrated a substantial link between DS and negative health impact [19-22,26,28,31,32,34,36,38,39].

Discussion

**Figure 2:**
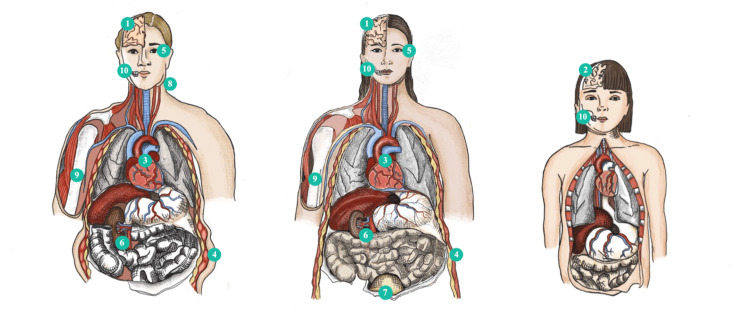
Representation of organs and systems affected by excess consumption of artificially sweetened beverages. This illustration is the authors' own creation. Numbers represent findings of studies that found a relationship between the consumption of artificially sweetened beverages and health impacts. 1: Mental health. 2: Child neurodevelopment. 3: Cardiac remodeling. 4: Obesity and metabolic syndrome. 5: Diabetic retinopathy. 6: End-stage renal disease. 7: Lower urinary tract symptoms. 8: Lymphoma and multiple myeloma. 9: Fractures. 10: Dental erosion.

Little is known about the impacts that ASBs pose on health, especially those used in beverages like DS. As discussed above, some findings have been made about their relation to cardiometabolic implications, but these findings remain controversial. As more research is being done on this subject, and with better quality, we set up to review any new discoveries or advances in terms of possible harms that these substances may generate acutely or chronically for consumers, especially findings not related to obesity, incident diabetes, and metabolic syndrome. We divided our research into several subtopics and analyzed the data. The findings can be visualized in Table 2 and Figure 2.

**Table 2:** Associations between diet soda consumption and health impacts found by various studies. LAD: left atrial dimension; LVM: left ventricular mass; BMI: body mass index; RA: rheumatoid arthritis; SSBs: sugar-sweetened beverages; LUTS: lower urinary tract symptoms; HbA1c: glycosylated hemoglobin; ESRD: end-stage renal disease; NHL: non-Hodgkin's lymphoma; BrAC: breath alcohol concentration; CVD: cardiovascular disease.

Author	Outcomes being studied in diet soda consumers	Year	Age of participants in years	Number of participants	Gender of participants	Results of the study	Significant negative health impact association found?	Type of study
Andersson et al. [19]	Cardiac remodeling	2015	X̄ = 55	n = 4,202	59% women, 41% men	Soda consumption, especially diet soda, was associated with higher LAD and LVM, compared to no soda consumption	Yes, with an identified confounder	Cohort study
Bragg et al. [32]	Eating disorders	2013	X̄ = 34	n = 2,077	87.2% women, 12.8% men	Individuals who consume any kind of soda regularly reported higher BMI and more eating psychopathology (eating disorders) than those who do not	Yes	Cross-sectional
Brown et al. [20]	Eating disorders	2013	Not reported	n = 397	Not specified	Individuals with bulimia nervosa consume excessive amounts of diet soda compared to non-eating disorder controls	Yes	Cohort study
Cohen et al. [21]	Child cognition	2018	Offspring follow-up at X̄ = 3.3	1,234 mother-child pairs, n = 2,468	Not specified	Maternal diet soda consumption during pregnancy may adversely impact child cognition	Yes	Cohort study
Guo et al. [38]	Depression	2014	X̄ = 61	n = 263,923	51% women, 49% men	Frequent consumption of sweetened beverages, especially diet drinks, may increase depression risk among older adults, whereas coffee consumption may lower the risk	Yes	Case-Control study
Fung et al. [22]	Hip fractures	2014	X̄ = 53	n = 73,572	100% women	Increased soda consumption of all types may be associated with an increased risk of hip fractures in postmenopausal women	Yes	Cohort study
Hatch et al. [23]	Fecundability	2018	X̄ = 30	n = 4,873	Not reported; 1045 couples, 161 men, and 2518 women who completed the food survey	Diet soda had little association with fecundability	No	Cohort study
Hu et al. [24]	Rheumatoid arthritis	2014	30-55 range	n = 79,570	100% women	Diet soda is not associated with an increased risk of seropositive RA in women, independent of other dietary and lifestyle factors	No	Cohort study
Leung et al. [33]	Cell aging	2014	X̄ = 40	n = 5,309	52% women, 48% men	No significant associations were observed between the consumption of diet sodas or non-carbonated SSBs and telomere length	No	Cross-sectional
Ma et al. [25]	Fatty liver disease	2015	X̄ = 50	n = 8,542	Not specified	Diet soda intake was not associated with measures of fatty liver disease	No	Cohort study
Maserejian et al. [26]	Lower urinary tract symptoms	2013	30-79 range	n = 5,502	61% women, 39% men	Women with recently increased soda intake, particularly caffeinated diet soda, had higher symptom scores, urgency, and LUTS progression	Yes	Cohort study
Mirghani et al. [34]	Diabetic retinopathy	2021	X̄ = 51	n = 200	49% women, 51% men	In diabetics, diet soda was associated with higher HbA1c and retinopathy, while non-caloric flavor consumption was associated with obesity	Yes	Cross-sectional
Park et al. [27]	Fatty liver disease	2021	X̄ = 63 (offspring), X̄ = 48 (3rd generation participants)	n = 1,636	58% women, 42% men	Diet soda was not associated with worsening liver fat	No	Cohort study
Rebholz et al. [28]	End-stage renal disease	2017	X̄ = 54	n = 15,368	55% women, 45% men	Diet soda consumption was associated with higher ESRD risk	Yes	Cohort study
Sakaki et al. [29]	Hypertension and hyperlipidemia	2022	X̄ = 11	n = 9,043	65% women, 35% men	Diet soda was not associated with either hypertension or hyperlipidemia risk in young adulthood	No	Cohort study
Samman et al. [35]	Caries	2022	21-60 range	n = 2,368	51% women, 49% men	High diet drinks consumption slightly increased the odds of dental erosion among US adults, although this relationship was not statistically significant	No	Cross-sectional
Schernhammer et al. [30]	Leukemia and lymphoma	2012	X̄ = 50	n = 97,334	61% women, 39% men	In men, >1 daily serving of diet soda increased risks of NHL and multiple myeloma in comparison with men who did not consume diet soda	No, the authors suggest they cannot rule out chance as the cause given the difference in both genders	Cohort study
Stamates et al. [39]	Breath alcohol concentrations	2015	X̄ = 23	n = 20	50% women, 50% men	Participants had significantly higher BrAC when the mixer was diet as compared to regular for both alcohol dose conditions	Yes	
Sumorok et al. [37]	Urinary lithogenicity	2012	18-65 range	n = 12	Not specified	The potential of diet sodas to reduce the recurrence of kidney stones does not appear to be great at ingested volumes of approximately 1 L per day	Neutral	Crossover study
Vyas et al. [31]	Risk of cardiovascular events	2015	X̄ = 61	n = 161,808	100% women	There is an association between high diet drink intake and CVD outcomes and mortality in post-menopausal women	Yes	Cohort study
Yu et al. [36]	Depression	2017	X̄ = 53	n = 18,838	69% women, 31% men	There is an association between depression and the consumption of sweeteners and diet drinks, which was more apparent among women than men	Yes	Cross-sectional

Mental Health

Mental health and its relationship with ASB consumption were studied by different authors [20,32,36,38]. Two of the studies focused on depression (36,38). One of them was cross-sectional [36], and the other one was a case-control study [38]. Both studies consisted of large groups of patients, with more than 18,000 participants. In both studies, the majority of the patients involved were women, and the design was based on a survey evaluating coffee consumption, ASBs consumption, and major depression. They found a statistically significant association between depression and consumption of ASB, which was more apparent among women than men. Interestingly, in the study of Guo et al., coffee was found to be protective against depression [38].

Another approach evaluated the consumption of these substances in the setting of eating disorders [20,32]. They were also both cross-sectional studies. DS drinkers were more likely to report binge eating and purging than regular soda drinkers, who were more likely to report these behaviors than non-soda drinkers [32]. Individuals with eating disorders, particularly bulimia nervosa, consumed more DS than controls. Eating disorder symptoms that reflect increased appetitive drive or increased weight concerns were associated with increased DS intake [20].

The findings of all these studies highlight the importance of monitoring DS intake in patients with mood and eating disorders and can help to further understand behavioral patterns in these patients.

Child Neurodevelopment

A prospective cohort study done by Cohen et al. explored the relationship between ASB and child cognitive development [21]. They followed 1,234 mothers’ diets during their pregnancies and years later monitored their children’s neurodevelopment. They found that maternal DS consumption during pregnancy may adversely impact child cognition, as children's cognitive scores were inversely proportional to mothers' SSB and ASB consumption. The results were statistically significant. Interventions and policies that promote healthier diets during pregnancy, including avoiding DS, may prevent adverse effects on childhood cognition. Further studies should focus on studying the neurological effects of ASBs and the mechanisms by which these substances impact cognition.

Cardiometabolic Impact

Our literature search only included results that focused on subjects other than obesity, incident diabetes mellitus, and metabolic syndrome, for reasons explained above.

However, we were able to find other forms of cardiovascular impact not previously studied before. The data revolving around cardiac remodeling [19], hypertension, and hyperlipidemia in adulthood after consumption of DS in childhood [29], and the risk of specific cardiovascular events [31] are scarce, so we included these studies in our review.

Andersson et al., in their cross-sectional study, measured cardiac remodeling in SSB and DS consumers [19]. Despite having identified the high body weight of soda drinkers as a confounder, they found that soda consumption, especially DS, was associated with higher left atrial dimension (LAD) and left ventricular mass (LVM), compared to no soda consumption [19]. Another study found that DS was not associated with either hypertension or hyperlipidemia risk in young adulthood in DS consumers [29], and other authors found an association between high diet drink intake and cardiovascular disease (CVD) outcomes and mortality in post-menopausal women [31].

These studies allowed two conclusions to be made: DS consumption in high quantities might increase not only metabolic risks but also cardiovascular risks, which should prompt clinicians to educate patients on the regulation of ASBs. On the other hand, it raises concerns that more studies should be conducted to further analyze the mechanisms in which these substances impact cardiometabolic risk, and to reproduce the data so results may be implemented in public health policies.

Diabetic Retinopathy

One article was found that studied the relationship between the consumption of DS and diabetic retinopathy in the diabetic population [34]. It found statistically significant results and determined that, in diabetics, DS consumption was associated with higher glycosylated hemoglobin (HbA1c) and retinopathy, while non-caloric flavor consumption was associated with obesity. This challenges the common myth that diabetics can consume DS without harm, and questions the fact that a healthy diet in this population is based solely on the glycemic charge of meals. New studies should be conducted to further understand the mechanisms of the worsening of retinopathy in these patients.

Urogenital Implications

Associations between DS and the genitourinary system have been made by several authors [26,28,37]. Rebholz et al. studied the incidence of end-stage renal disease (ESRD) in consumers of the beverage [28]. They designed a prospective cohort study, in which participants were asked about DS consumption habits and were followed over time. Over a median follow-up of 23 years, 357 incident ESRD cases were observed. Relative to one glass/week of DS, consuming one to four glasses/week, five to seven glasses/week, and seven glasses/week, respectively, was associated with 1.08-times, 1.33-times, and 1.83-times higher risk of ESRD after adjusting multiple variables. The results were statistically significant. Further research is necessary to validate these findings in other study populations and to examine potential mechanisms through which DS could impact kidney disease.

On the other hand, orange DS was believed to decrease urinary lithogenicity by increasing urine citrate excretion. A study was conducted in which participants consumed orange DS three times a day besides their regular diet to evaluate if lithogenicity changed with the consumption of this substance [37]. They then served as their own controls. Diet orange soda increased urinary citrate excretion by 60 mg/day, which was not statistically significant. There was no significant change in pH from the control period to the study period. Urine volumes and creatinine excretions were not significantly different between the control and study periods.

Another cohort study explored the effect that ASBs have on lower urinary tract symptoms (LUTS) [26]. With statistically significant results, they found that caffeinated ASBs increased LUTS in the women’s cohort, and that citrus juice consumption diminished them in the men’s cohort. Findings support recommendations to limit caffeinated beverage intake for LUTS, and in men, they suggest the benefits of citrus juice consumption. Further clinical research is warranted, particularly on the precise role of sodas containing artificial sweeteners in bladder sensations and urological function.

Carcinogenesis

A prospective cohort study conducted with over 90,000 patients studied the possibility of carcinogenic properties of DS [30]. In men, > one daily serving of DS increased risks of non-Hodgkin's lymphoma and multiple myeloma in comparison with men who did not consume DS. However, even if the results in the study were statistically significant, the authors specified that they cannot rule out chance as the cause given the difference in findings in both genders.

Fatty Liver Disease

Two studies were found that evaluated the relationship between DS consumption and fatty liver disease incidence [25,27]. With more than 8,000 patients studied in total, both studies agree that DS intake was not associated with measures of fatty liver disease or worsening liver fat.

Autoimmune Disease

Increased risk of different chronic inflammatory diseases, such as type 2 diabetes and CVDs, have been recurrently associated with ASBs [24]; nevertheless, the relationship between ASB and rheumatoid arthritis (RA), the most common autoimmune inflammatory disease, remains uncertain. Hu et al. conducted a prospective cohort study evaluating the tendency of soda consumption and risk of developing RA in women [24]; following 79,570 women between 1980 and 2008, and 107,330 women between 1991 and 2009. Follow-up was made every four years, obtaining information from a validated food-frequency questionnaire, and finally, incident RA cases were validated by medical record review. The results showed a significant association between SSB consumption and increased risk of RA in the first cohort, but not in the second cohort; on the other hand, neither of the two cohorts showed a remarkable association between DS consumption and risk of RA. Advanced investigations are required to corroborate and fully exclude the possible risk of RA caused by DS.

Hip Fractures

In a large cohort study conducted by Fung et al., [22], with a total of 73,572 female patients, it was determined that, after repeatedly assessing patients every four years for DS consumption and incident risk fractures, increased soda consumption of all types may be associated with increased risk of hip fracture in postmenopausal women. The risk was significantly elevated in consumers of both regular soda (RR: 1.19; 95% CI: 1.02, 1.38) and DS (RR: 1.12; 95% CI: 1.03, 1.21) and also did not significantly differ between colas and non-colas or sodas with or without caffeine. A clear mechanism was not apparent on the basis of these observational data and should be further studied.

Other Health Impacts

A prospective cohort study was done among 3,828 women planning pregnancy and 1,045 of their male partners, evaluating the association of ASB intake with fecundability; following enrolled participants for up to 12 menstrual cycles or up to pregnancy [23]. Outcomes indicate that both female and male intakes of SSB were associated with reduced fecundability; however, DS had little association with fecundability.

Leung et al. examined, through a cross-sectional study, the associations between ASB and telomere length in a sample of 5,309 healthy adults [33]. It was concluded that SSB consumption was associated with shorter telomeres, influencing accelerated cell aging; nevertheless, no significant associations were observed between the consumption of DS and telomere length.

The effect of DS on the chemical loss of mineralized tooth substance was measured by a cross-sectional study that involved 2,368 male and female participants [35]. The results revealed that high consumption of DS slightly increased the odds of dental erosion, although the relationship was not statistically significant. To understand more about dental erosion risk factors, more research is needed.

A randomized controlled trial, done on 20 participants and using two different moderate alcohol doses [39], confirmed the premise that mixing alcohol with diet beverages can result in higher breath alcohol concentrations (BrAC) when compared with mixing the same amount of alcohol with sweetened beverages. Even though diet mixers may reduce caloric intake, they increase the harm associated with higher BrAC.

*Evaluation of Bias of Selected Studies*

The studies included in our final synthesis may have incurred potential information biases, mainly due to the way in which they measured the results, with a possible overestimation of the impact on the quality of life of excess ASB use in the long term. The inter-study variability in the assessment of outcomes and mainly the lack of blinding, both for the assessor and the patients, which are very frequent in these studies, could lead to results with a magnitude greater than the real one. However, what would probably occur in other more controlled settings is a possible decrease in the observed effect, but maintaining an impact that continues to be clinically relevant.

As a result of the synthesis, the overall quality of the included studies was classified as moderate, which can be corroborated in an analysis of the biases according to the qualitative score of the Risk of Bias In Non-randomized Studies of Interventions (ROBINS-I) bias assessment tool [40], available in Table 3. This determination is due to the product of possible errors, most of them not severe and none of them critical. These errors were related especially to aspects that contributed to assessing the importance of the findings, such as clinical relevance, and measurement of patient-centered outcomes (quality of life, safety, and time and quality of follow-up) of some studies that were concerned with medium- or long-term sustainability.

**Table 3:** ROBINS-I tool for the evaluation of bias. ROBINS-I: Risk of Bias In Non-randomized Studies of Interventions.

Author	Confounding	Selection of participants	Classification of interventions	Deviation from intended interventions	Missing data	Measuring outcomes	Selection of the reported result	Overall
Andersson et al. [19]	Moderate	Moderate	Low	Low	Low	Moderate	Low	Moderate
Bragg et al. [32]	Low	Low	Low	Low	Low	Low	Low	Low
Brown et al. [20]	Low	Low	Low	Low	Low	Low	Low	Low
Cohen et al. [21]	Serious	Moderate	Low	Low	Low	Serious	Low	Serious
Guo et al. [38]	Low	Moderate	Low	Low	Low	Low	Low	Moderate
Fung et al. [22]	Moderate	Moderate	Low	Low	Low	Moderate	Low	Moderate
Hatch et al. [23]	Moderate	Moderate	Low	Moderate	Moderate	Moderate	Low	Moderate
Hu et al. [24]	Moderate	Moderate	Low	Low	Low	Moderate	Low	Moderate
Leung et al. [33]	Serious	Moderate	Low	Low	Low	Serious	Low	Serious
Ma et al. [25]	Low	Moderate	Low	Low	Low	Low	Low	Moderate
Maserejian et al. [26]	Low	Moderate	Low	Low	Low	Low	Low	Moderate
Mirghani et al. [34]	Moderate	Moderate	Low	Low	Low	Moderate	Low	Moderate
Park et al. [27]	Low	Moderate	Low	Low	Low	Low	Low	Moderate
Rebholz et al. [28]	Low	Moderate	Low	Low	Low	Low	Low	Moderate
Sakaki et al. [29]	Low	Moderate	Low	Low	Low	Low	Low	Moderate
Samman et al. [35]	Low	Moderate	Low	Low	Low	Low	Low	Moderate
Schernhammer et al. [30]	Serious	Moderate	Low	Low	Low	Serious	Low	Serious
Stamates et al. [39]	Low	Moderate	Low	Low	Low	Low	Low	Moderate
Sumorok et al. [37]	Low	Moderate	Low	Low	Low	Low	Low	Moderate
Vyas et al. [31]	Low	Moderate	Low	Low	Low	Low	Low	Moderate
Yu et al. [36]	Low	Low	Low	Low	Low	Low	Low	Low

Limitations

Possible limitations of our study include a scarcity of data on the specific organ systems, and variability in the quality of data gathered. Further studies should delve further to better understand the pathophysiologic mechanisms of these associations.

## Conclusions

The question of whether ASBs such as DS are a preferred substitute is becoming more and more important in terms of public policy due to mounting evidence of the potential negative health effects of their excessive consumption. This systematic review, the first of its kind to our knowledge, sheds light on how excessive DS consumption can affect multiple organ systems. The excessive consumption of these beverages does indeed pose a threat to health, as it impacts many organs and systems.

Some of the risks are listed as follows: mental health (including incidental mood disorders such as depression and eating disorders such as bulimia); delays in child neurodevelopment when consumed by mothers during pregnancy; cardiac remodeling (increased LAD and LVM); evidence of worsening retinopathy as well as increased HbA1c in diabetics; increased urinary symptoms in women; incidental ESRD, a modest association between consumption of ASB and non-Hodgkin's lymphoma as well as multiple myeloma in men, incidental rheumatoid arthritis in women; increased risk of hip fractures in postmenopausal women; incidental dental erosion and caries; and finally, increases in BrAC when used in alcoholic beverages.
