# Correction: Artificially Sweetened Beverages Beyond the Metabolic Risks: A Systematic Review of the Literature

**DOI:** 10.7759/cureus.c262

**Published:** 2025-08-26

**Authors:** Tomas Escobar Gil, Juanita Laverde Gil

**Affiliations:** 1 Medicine, Universidad CES, Medellín, COL; 2 Medicine, Universidad CES, Medellin, COL

This article has been corrected to fix the following typos in the PRISMA diagram:

- "Records marked as ineligible by automation tools" has been corrected from 0 to 476. This changes "Records removed before screening" from 249 to 725.
- "Records identified from databases" has been updated to list all databases.

